# Light‐Induced Welding of Electrospun Poly(*ε*‐caprolactone) Nanofibers in a Nonwoven Mat by Leveraging the Photothermal Effect of Gold Nanocages

**DOI:** 10.1002/marc.202401144

**Published:** 2025-03-19

**Authors:** Haoxuan Li, Yidan Chen, Tong Wu, Wenxia Wang, Haoyan Cheng, Jiajia Xue, Younan Xia

**Affiliations:** ^1^ The Wallace H. Coulter Department of Biomedical Engineering Georgia Institute of Technology and Emory University Atlanta GA 30332 USA; ^2^ State Key Laboratory of Advanced Fiber Materials College of Materials Science and Engineering Donghua University Shanghai 201620 P. R. China; ^3^ School of Materials Science and Engineering Georgia Institute of Technology Atlanta GA 30332 USA; ^4^ School of Chemistry and Biochemistry School of Chemical and Biomolecular Engineering Georgia Institute of Technology Atlanta GA 30332 USA

**Keywords:** electrospinning, nanofibers, gold nanocages, photothermal paper, welding

## Abstract

Nonwoven mats of electrospun nanofibers are widely used in an array of applications, including those related to filtration, textiles, and tissue engineering. The performance of the mats is often plagued by their relatively weak mechanical strength due to the lack of bonding at the junction points between fibers. To address this issue, here a controllable technique is demonstrated for welding a nonwoven mat of poly(*ε*‐caprolactone) fibers into an interconnected network by leveraging the photothermal effect of Au nanocages under the irradiation of a near‐infrared laser. Upon irradiation for 2 s only, the poly(*ε*‐caprolactone) fibers in a nonwoven mat are permanently welded at the junction points. When the irradiation time is increased to 5 s, the fibers fused together transforming the porous and opaque mat into a transparent solid film. In addition to strengthening nonwoven mats of electrospun nanofibers, this technique may open the door to new applications such as masking, patterning, and printing.

## Graphical abstract

The light‐induced heating enabled by Au nanocages offers a simple and effective method for selectively welding poly(*ε*‐caprolactone) nanofibers at their junction points, reinforcing the mechanical property of a fibrous mat. By controlling the extent of welding, the fibrous mat can be transformed into a solid film with high transparency, expanding the scope of applications.

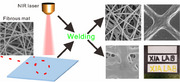

## 1 Introduction

Electrospun nanofibers have found widespread application in a wide variety of areas, including biomedicine, filtration, textiles, and packaging, owing to their unique characteristics such as high porosity, large specific surface areas, and tunable diameters ranging from nanometers to micrometers.^[^ ^1^, ^2^, ^3^ ^]^ The nanofibers are typically collected and utilized as nonwoven mats without inter‐fiber bonding, resulting in compromised mechanical properties.^[^ ^4^ ^]^ To address this issue, methods such as conventional thermal heating,^[^ ^5^, ^6^ ^]^ light‐induced heating,^[^ ^7^, ^8^ ^]^ chemical cross‐linking,^[^ ^9^, ^10^, ^11^ ^]^ and solvent vapor treatment^[^ ^12^, ^13^, ^14^, ^15^ ^]^ have been explored to weld the nanofibers at their junction points. Despite their effectiveness in achieving welding fibers at their cross points and enhancing mechanical properties, these techniques typically weld the entire mat rather than at specific regions, limiting their scope of applications. To this end, light‐induced heating based upon the photothermal effect would offer some immediate advantages, including the capability to achieve rapid and localized heating. This capability would rapidly increase the local temperature in a fibrous mat, offering a promising approach to welding the nanofibers in a nonwoven mat with precise spatial and temporal controls.^[^ ^16^ ^]^ In prior studies, Kaner and coworkers,^[^ ^7^ ^]^ as well as Garnett and coworkers^[^ ^8^ ^]^ leveraged the photothermal effect of polyaniline nanofibers and silver nanowires, respectively, to weld their films in an area‐selected fashion. However, conventional polymers cannot utilize light for heat generation due to their weak absorption of visible and near‐infrared (NIR) light.

To address the issue of light absorption, an optical absorber has been directly incorporated into electrospun nanofibers to serve as a photothermal agent for light‐induced welding. Our group previously reported the successful incorporation of an organic dye, indocyanine green (ICG), into poly(*ε*‐caprolactone) (PCL) nanofibers during the electrospinning process.^[^ ^17^ ^]^ Under the irradiation of an 808‐nm diode laser at 0.4 W cm^−2^ for just 2 s, the temperature of the ICG‐doped PCL fibrous mat could be increased to ≈40 °C, resulting in welding at the junction points of the fibers. In this case, ICG had to be used at a level as high as 1 wt.% in the nanofibers and the resulting fibrous mat exhibited a bright green color due to the high loading level of ICG. This can be attributed to the small absorption cross‐section of ICG and the low photothermal efficiency of the fibers, making it necessary to enhance the photothermal performance of the fibrous mat by increasing the ICG content. To improve the photothermal efficiency, Li and coworkers^[^ ^18^ ^]^ switched to nanofibers with a core‐sheath structure, with the core phase containing a mixture of olive oil and 4,7‐bis(4‐(bis(4‐(octyloxy)phenyl)amino)phenylbenzo[1,2‐c:4,5‐c'lbis[1,2,5]thiadiazole (BPBBT) molecules and a sheath comprised of poly(vinylidene fluoride‐*co*‐hexafluoropropylene) (PVDF‐HFP). In this configuration, the content of BPBBT in the nanofibers was only 0.67%, but the mat of core‐sheath nanofibers could achieve a temperature rise to ≈56 °C under 1 sun irradiation. It was argued that this design significantly enhanced the intermolecular motion of organic dyes, promoting non‐radiative energy dissipation. However, the structural complexity of the nanofibers makes it challenging to fabricate the fibrous mat on a large scale. In a different approach, our group switched from organic dyes to noble‐metal nanostructures such as Au nanocages (AuNCs) to leverage their localized surface plasmon resonance (LSPR) properties and thus extraordinarily large absorption cross‐sections. Featuring a hollow interior, porous walls, and controllable absorption peaks in the range of 400–1200 nm, AuNCs have emerged as a class of attractive photothermal agents for applications requiring localized heating. In one study, we demonstrated the temperature of a nonwoven mat of polyvinylidene fluoride (PVDF) nanofibers could be rapidly increased to 73.9 °C under the irradiation of an 808‐nm diode laser at 0.2 W cm^−2^ for 60 s although it only contained 0.01 wt.% AuNCs.^[^ ^19^ ^]^ This remarkable increase in temperature can be attributed to the extraordinarily‐large (about one million times of a typical organic dye molecule) absorption cross‐section of AuNCs and their high photothermal conversion efficiency.

In this study, we explore the use of AuNCs for welding the nanofibers in a nonwoven mat owing to their outstanding performance as a photothermal agent.^[^ ^20^, ^21^ ^]^ Incorporating AuNCs into nanofibers does not alter the appearance of the fibrous mat, particularly its color, due to its use at an extremely low level. The core innovation of this work lies in the utilization of AuNCs as a more effective photothermal converter, achieving 100‐fold reduction in the loading of photothermal agent while preserving the original color of the pristine PCL nanofibrous mat. When incorporated into polymer nanofibers, AuNCs can absorb light to generate localized heating, facilitating the welding of nanofibers at their junction points upon light irradiation. By increasing the irradiation time and/or the light power density, the nanofibers can be over‐welded to eliminate the pores in the mat,^[^ ^14^ ^]^ transforming the opaque sample into a transparent, solid film. This phenomenon can be leveraged to develop a new type of thermal paper, eliminating the need of bisphenol A (BPA), a hazardous compound widely used as a chemical developer in the current design of thermal paper.

## 2 Results and Discussion

**Figure 1:**
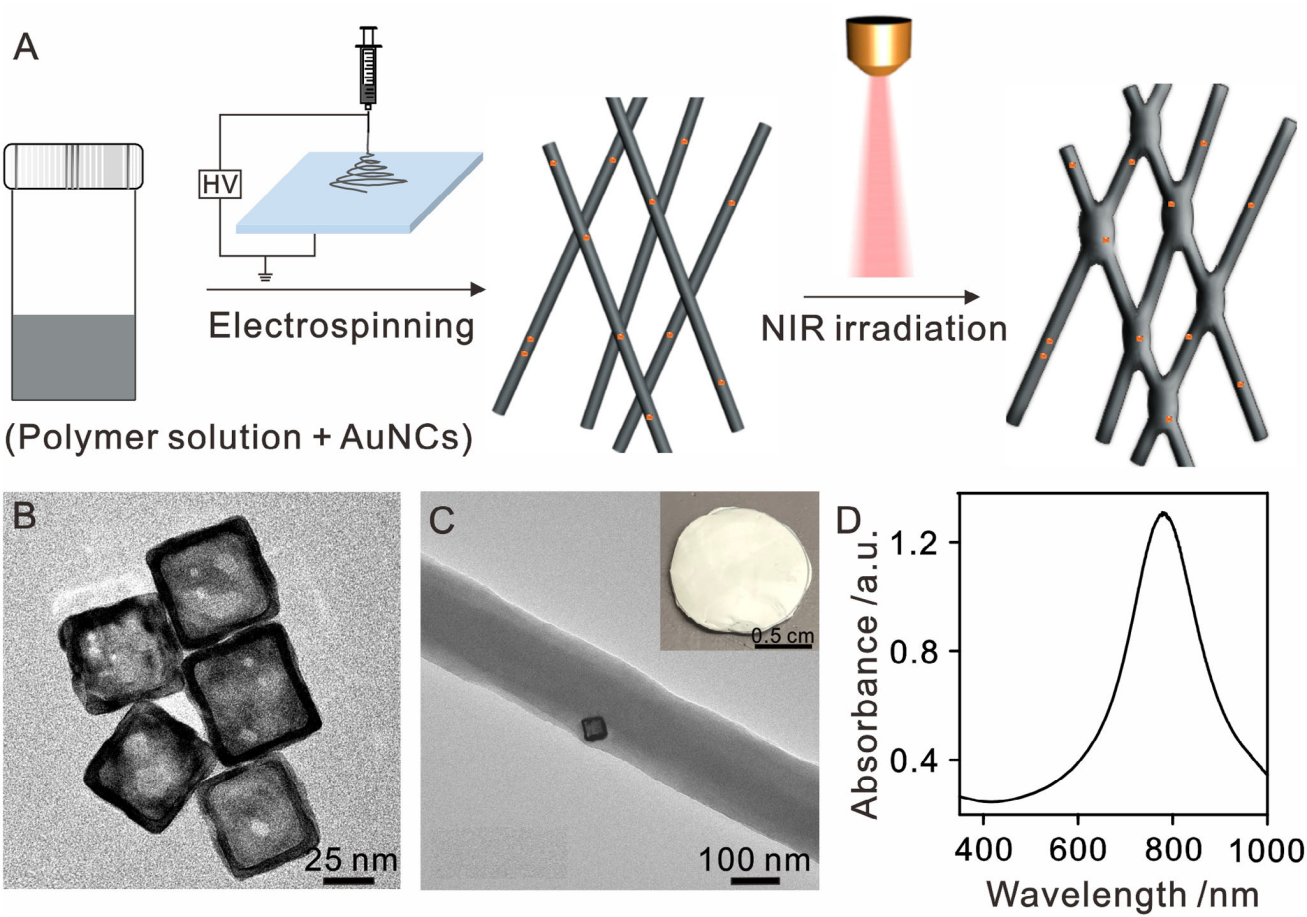
A) Schematic illustration of light‐induced welding of a nonwoven mat of polymer nanofibers containing AuNCs, B) TEM image of the AuNCs, C) TEM image of a segment of the PCL nanofiber containing a AuNC (inset: a digital photograph of the mat), and D) UV–vis–NIR spectrum recorded from an aqueous suspension of the AuNCs.

**Figure** **1**A shows a schematic of our experimental set‐up and illustrates the light‐induced welding process. The nonwoven mat of polymer nanofibers containing AuNCs was fabricated by electrospinning, a simple and versatile technique capable of producing fibers with diameters ranging from tens of nanometers to a few micrometers. In this study, PCL was selected as a model polymer due to its relatively low melting point ≈55–60 °C. We investigated PCL nanofibers with three AuNCs loadings, which were determined to be 0.004, 0.018, and 0.037 wt.%, respectively, by inductively coupled plasma mass spectrometry (ICP‐MS) analysis (Table S1, Supporting Information). As shown in Figure 1B, the AuNCs exhibit a well‐defined hollow interior, together with an average outer edge length of ≈53 nm. Figure 1C demonstrates that the incorporation of AuNCs did not cause any major changes to the electrospinning process, and the mat comprised of AuNCs‐loaded nanofibers showed a light blue color different from the white color typically displayed by a mat of plain PCL nanofibers (Figure S1, Supporting Information). The UV–vis–NIR spectrum in Figure 1D indicates that the absorption peak of the AuNCs (in water, with a dielectric constant slightly lower than that of PCL) was located at ≈800 nm, in proximity to the wavelength (808 nm) of the diode laser to be used for photothermal welding. Notably, following incorporation into the PCL nanofibers, the absorption peak of the AuNCs remained at ≈800 nm due to the passivation of their surface by polyvinyl pyrrolidon (PVP) (Figure S2, Supporting Information). In a typical welding process, the nonwoven mat containing AuNCs was exposed to the diode laser at an optimized power density for different periods of time. The samples before and after NIR irradiation, in particular, the junction points between the nanofibers, were analyzed by means of scanning/transmission electron microscopy (S/TEM).

**Figure 2:**
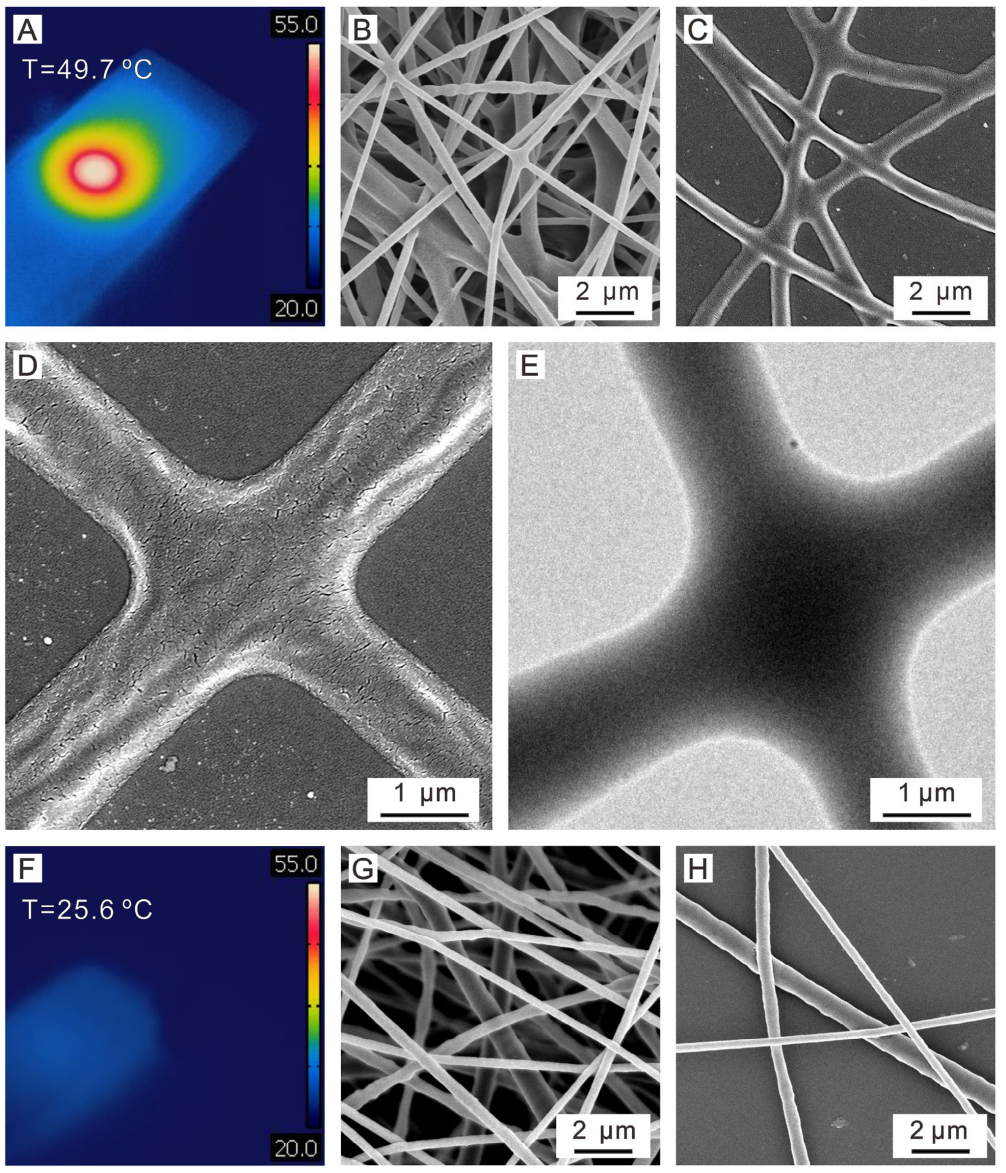
A) Infrared picture taken from a fibrous mat containing AuNCs (0.018 wt.%) under irradiation with an 808‐nm laser for 2 s at a power density of 0.8 W cm^−2^. B,C) SEM images of the sample after the irradiation. D,E) SEM and TEM images of a junction point between two fibers after the irradiation, and F–H) infrared and SEM images taken from a control sample made of plain PCL nanofibers when irradiated under the same condition.

As a cost‐effective light source, NIR diode lasers have garnered significant interest for applications such as controlled drug release, phototherapy, and micropatterning.^[^ ^22^, ^23^, ^24^ ^]^ Upon exposure to the diode laser, the AuNCs can absorb the incident light and have it effectively converted to heat through the photothermal effect. Given that the fibers have a better thermal conductivity relative to the surrounding air, the generated heat can rapidly transport along the fibers. At the junction points, the heat from different fibers converges, resulting in a higher temperature at these locations compared to other regions of the fibers. Consequently, the PCL nanofibers can be preferentially softened and/or melted at the junction points, causing fusion or welding at these points. By optimizing the laser power density and/or duration of exposure, one can only weld the nanofibers at their junction points while sparing the other segments. As shown in **Figure** **2**A and the supplementary video 1, the surface of the fibrous mat could reach an overall temperature of ≈50 °C within 2 s of irradiation when the diode laser was set at a power density of 0.8 W cm^−2^. Figure 2B–E shows SEM and TEM images of a typical sample obtained after 2 s of irradiation. During the irradiation process, welding occurred exclusively at the junction points among the fibers, with minimal alterations to the topology and geometry of the fibers away from these junctions. Unlike conventional heating and vapor treatment, photothermal heating allows for spatial control to manipulate the locations of welding on the fibrous mat.^[^ ^7^ ^]^ In other words, the site of welding on the fibrous mat can be maneuvered by controlling the irradiation spot. As an effective photothermal agent, AuNCs play a crucial role in the NIR‐induced welding process. Figure 2F shows the temperature of a PCL fibrous mat fabricated using the same protocol but without including AuNCs. In this case, no temperature rise was detected when irradiated for 2 s, and the surface remained at ≈25.5 °C. In the absence of a photothermal agent, the pristine PCL nanofibers did not absorb light produced from the NIR laser, keeping the fibers as a loosely‐stacked nonwoven mat involving no welding at the junction points (Figure 2G,H). Besides, Fourier transform infrared (FT‐IR) spectra collected form the AuNCs/PCL nanofibrous mat before and after welding exhibited the same patten of peaks, suggesting that the welding induced by laser irradiation was a physical process, having essentially no impact on the chemical structure of the polymer (Figure S3, Supporting Information). Quantitative comparative analysis of laser‐welded (808 nm) versus pristine AuNCs/PCL nanofibrous mats revealed a remarkable tensile strength enhancement (16.19 ± 0.59 MPa vs 6.72 ± 0.96 MPa, see Figure S4, Supporting Information). This mechanical reinforcement arises from laser‐induced fusion at the fiber cross‐junctions, which effectively suppresses inter‐fiber slippage through polymer chain entanglement.

An interesting phenomenon was observed when the irradiation time was increased to 5 s at a power density of 0.8 W cm^−2^. The welding became so pronounced that all the fibers began to fuse together, resulting in the formation of a solid film. The transmittance of the fibrous mat also increased with prolonged irradiation time (Figure S5, Supporting Information). Different from conventional heating, light‐induced welding does not significantly alter the overall structure of the fibrous mat except at the irradiation sites. The area‐selective welding enables new applications related to writing or patterning on a mat made of electrospun nanofibers. A similar phenomenon was also observed when the fibrous mat was exposed to the laser at 1.2 W cm^−2^ for 3 s only, indicating a more rapid response to laser irradiation at a higher power density (Figure S6, Supporting Information). It is well‐established that the generated heat increases with the increase of power density, leading to the reduction in time required for fusion into a transparent film. In general, the response time of a fibrous mat containing AuNCs to NIR irradiation can be shortened by increasing the laser power density. In this paper, the term “response time” refers to the duration required for the porous mat to fuse into a solid, transparent film under laser irradiation. Additionally, we have analyzed the fibrous structures situated at the periphery, boundary, and center of the irradiated region. Our results demonstrate that the extent of fiber fusion diminishes progressively from the center of irradiation toward the outer regions (Figure S7, Supporting Information). This trend can be ascribed to the thermal gradient, characterized by higher heat concentration at the irradiation center, which gradually decreases in the outward direction. To study the effect of AuNCs content on fiber diameter and welding efficiency, we fabricated AuNCs/PCL nanofibers with different AuNCs loadings (0.004, 0.018, and 0.037 wt.%) by optimizing the electrospinning protocol. As the AuNCs content was increased from 0.004 to 0.018 and 0.037 wt.%, the diameter of the fibers increased slightly from 308.7 ± 65.5 to 363.8 ± 165.5 and 433.7 ± 173.7 nm and the response time decreased with increasing loading, indicating enhanced welding efficiency (Figure S8, Supporting Information). The higher density of AuNCs in the irradiated area promoted localized heat generation, thereby intensifying molecular chain interdiffusion at the fiber junctions. Notably, even when the content of AuNCs is reduced to 0.004 wt.%, the fiber membrane can still be completely welded by 808‐nm laser. To evaluate material stability, the AuNCs/PCL nanofibrous mats were subjected to accelerated aging under continuous 313‐nm UV laser irradiation in a controlled environmental chamber for two weeks. Remarkably, the aged samples maintained comparable mechanical properties to pristine mats (Figure S6, Supporting Information). This finding demonstrates the excellent long‐term stability of the AuNCs/PCL nanofibrous mats under operational storage conditions.

**Figure 3:**
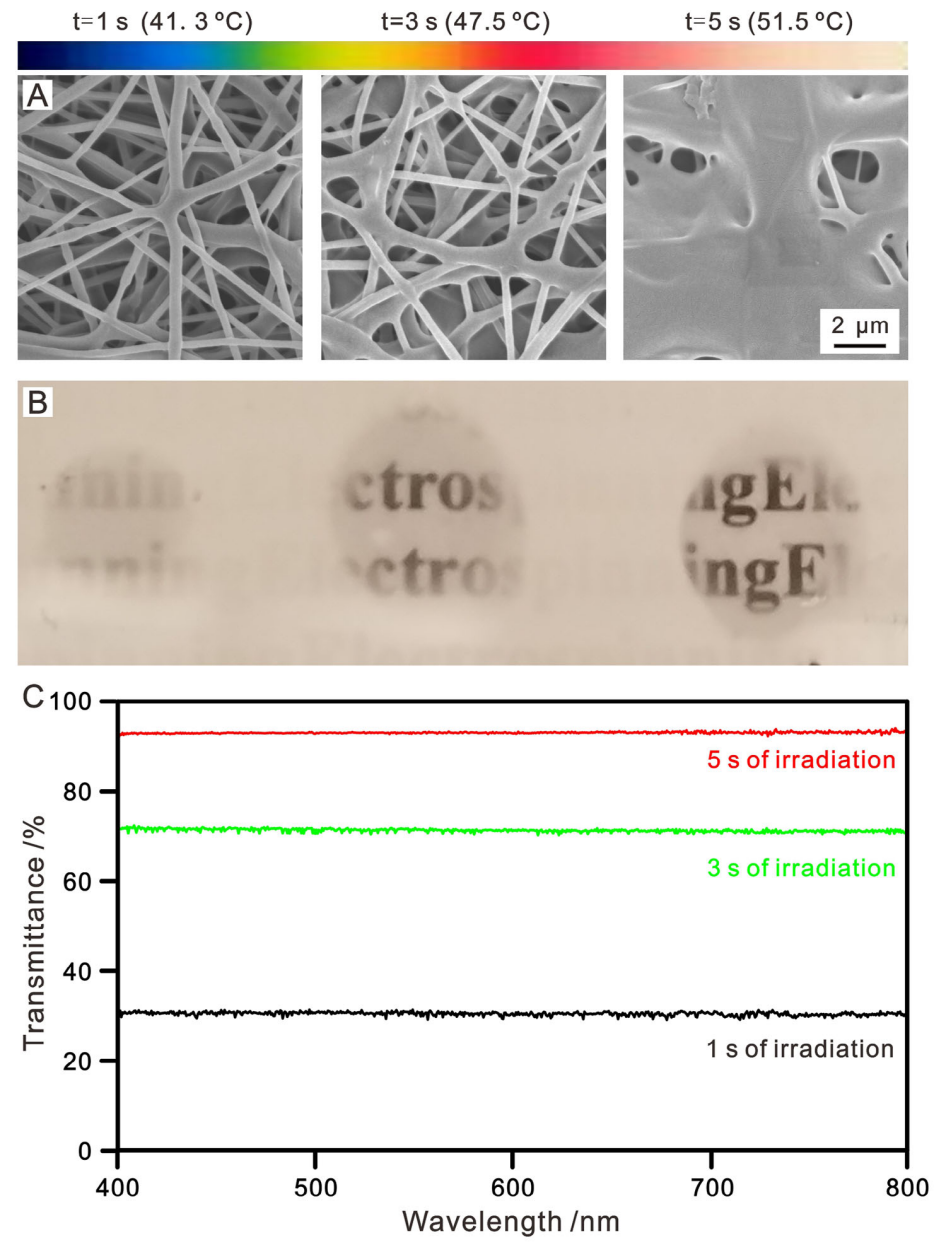
A,B) SEM images and photographs showing a fibrous mat containing AuNCs (0.018 wt.%) when irradiated by an 808‐nm laser at a power density of 0.8 W cm^−2^ for 1, 3, and 5 s, respectively. C) Transmittance spectra recorded from regions irradiated by the laser at a power density of 0.8 W cm^−2^ for 1, 3, and 5 s, respectively.

**Figure** **3** shows the transmittance (T) versus irradiation time for a fibrous mat containing AuNCs under the irradiation of the 808‐nm laser at a power density of 0.8 W cm^−2^. The low transparency of the pristine sample can be attributed to the scattering of light by the fiber/air interface associated with the pores, whose dimensions are comparable to the wavelength of visible light.^[^ ^25^, ^26^, ^27^ ^]^ The transmittance can be increased by filling the pores in the fibrous mat with a material bearing a refractive index on par with that of PCL. Such a filling significantly reduces light scattering from the interface, enabling the fabrication of an optically transparent film comprised of electrospun nanofibers. For example, Lee and coworkers demonstrated that the infiltration of pores in a fibrous mat with poly(dimethylsiloxane) (PDMS) could reduce light scattering, resulting in the formation of a transmittance film.^[^ ^28^ ^]^ Alternatively, we found that the pores could be eliminated by over‐welding the ICG‐loaded nanofibers in a nonwoven mat, albeit the high loading of ICG tended to tint the final film with a strong green color.^[^ ^14^ ^]^ By switching to AuNCs, it was feasible to make the over‐welded film highly transparent. As shown in Figure 3A, the surface temperature of the fibrous mat containing AuNCs increased from 47.5 to 56.8 °C when the irradiation time was increased from 1 to 5 s. As a result, the pores inside the fibrous mat completely disappeared, transforming the mat from an opaque to a transparent solid film (Figure 3B). Accordingly, the transmittance increased from 30.5 to 92.7% as the irradiation time was extended from 1 to 5 s (Figure 3C). We also fabricated AuNCs‐doped fibrous mats with different thicknesses by adjusting the duration of collection. After 1, 2, and 3 h of collection, fibrous mats with thicknesses of 112.8 ± 15.9, 169.5 ± 13.7, and 239.8 ± 17.0 µm, respectively, were obtained. As shown in Figure S9 (Supporting Information), the response time decreased with increasing fibrous mat thickness, possibly due to the increased number of AuNCs in the irradiated spot. Particularly, 3 s of irradiation on the 3 h‐electrospun mat achieved a similar level of welding to 5 s on the 2 h‐electrospun mat. Altogether, this experiment indicates that a diode laser can serve as a writing tool to create patterned features such as transparent circles and stripes in an opaque film (Figure S10, Supporting Information).

The light‐induced change in transparency can be potentially utilized to fabricate thermal paper, which is widely used for receipts, tickets, tags, and labels. Traditionally, a direct thermal printer is used to create images by heating a specially‐designed paper that contains a mixture of a leuco dye and an acidic developer. Upon heating, the dye will be protonated by the developer, resulting in a color change from white to black. The chemical developer used in the current thermal paper is based on BPA, an endocrine disruptor that can pose significant environmental and health risks.^[^ ^29^, ^30^ ^]^ It is worth noting that in recent years, no major progress has been made in the development of alternative color developers for thermal papers. At the moment, about half (49.2%) of the commercial products contain BPA as a functional developer in 311 randomly collected thermal papers from the German market.^[^ ^31^ ^]^ Experiments have shown that BPA can penetrate the skin to a depth that makes it impossible to wash off. Therefore, individuals who repeatedly handle thermal printer papers for 10 h per day, such as cashiers, may be exposed to BPA at a dose of 71 µg day^−1^, which is 42 times the tolerable daily intake (TDI).^[^ ^32^ ^]^ There is a pressing need to develop simple and highly sensitive thermal papers that eliminates the involvement of BPA.

**Figure 4:**
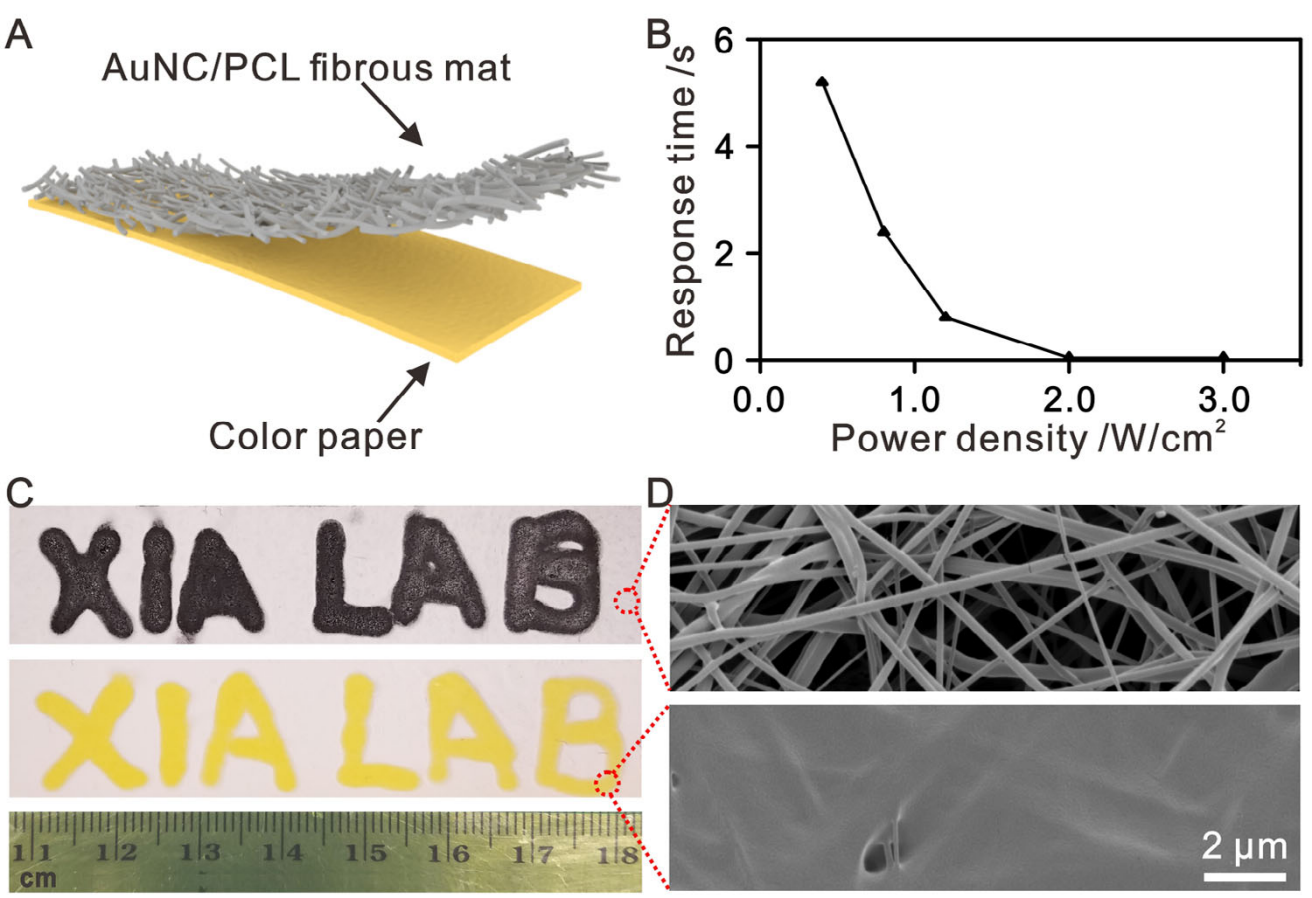
A) Schematic of photothermal paper based upon a nonwoven mat of electrospun nanofibers containing AuNCs (0.018 wt.%). B) Plot of the response time as a function of the power density of the diode laser. C) Letters written with a diode laser on the fibrous mats containing AuNCs supported on black and yellow papers, respectively. D) SEM images take from a region of the mat before (upper) and after (lower) the irradiation by a diode laser.

To differentiate from the thermal papers involving conventional heating and a chemical developer, we call our proposed product photothermal paper. As shown in **Figure** **4**A, the photothermal paper can be fabricated by coating the surface of a conventional color paper (those in black and yellow colors were chosen for this study) with a fibrous mat containing AuNCs. The as‐fabricated mat appeared almost white in color due to strong light scattering from the fiber/air interface at the pores, completely blocking the color displayed by the paper underneath. Upon irradiation with a diode laser, the AuNCs generate localized heating, causing the fibers in the irradiated area to fuse into a transparent solid film, thereby revealing the color of the underlying paper. At the same time, the fibers outside the irradiated area remained in their original porous structure and retained the white color. As shown in Figure 4B, the response time decreased from 5.3 to 0.87 s, as the power density was increased from 0.4 to 1.2 W cm^−2^. Significantly, when the power density was increased to 2.0 W cm^−2^, the response time was shortened to 0.05 s, which is sufficient to meet the requirements for thermal paper. Furthermore, the melting point of PCL is ≈60 °C, indicating that thermal paper based on PCL fibrous mat should exhibit good stability and can be stored for extended periods. For applications requiring higher temperatures, polymers with higher melting points can be selected. In a proof‐of‐concept, the efficient light‐induced welding was utilized to fabricate patterned transparent features in an opaque fibrous mat. As shown in Figure 4C, the phrase “XIA LAB” can be readily hand‐inscribed using a diode laser on the photothermal paper. The SEM images taken from different regions of the thermal paper further confirmed that the transformation from opaque to transparent was achieved by over‐welding the fibrous structure (Figure 4D). We believe that this chemical‐free, rapid‐response, and biodegradable photothermal paper has significant potential for use in printing, recording, and commercial labeling.

## 3 Conclusion

In summary, we have demonstrated a simple and effective method for selectively welding polymer nanofibers at their junction points by leveraging the localized heating generated by AuNCs. Due to their extremely large absorption cross‐section and the excellent photothermal conversion efficiency, AuNCs can be used at a loading content as low as 0.004 wt.% to cause essentially no alteration to the appearance of the fibrous mat, particularly its color. The welding process can be precisely controlled, given its strong dependence on the irradiation position, duration, power intensity, and spot size of the NIR diode laser. In addition to the fabrication of the fibrous mat with enhanced mechanical properties, the nanofibers containing AuNCs can also be readily over‐welded to transform the fibrous mat into a transparent solid film, opening the door to new applications such as masking, patterning, and printing.

## Conflict of Interest

The authors declare no conflict of interest.

## Supporting information



Supporting Information

Supplemental Video 1

## Data Availability

The data that support the findings of this study are available from the corresponding author upon reasonable request.
