# Supplementary material for: Light‐Induced Welding of Electrospun Poly(ε‐caprolactone) Nanofibers in a Nonwoven Mat by Leveraging the Photothermal Effect of Gold Nanocages
Source: Macromol Rapid Commun. 2025 Mar 19;46(13):2401144. doi: 10.1002/marc.202401144 (PMC12227221; doi:10.1002/marc.202401144)
Supplement: Supplementary file 1 — Supporting Information [file MARC-46-2401144-s002.docx]

**Supporting Information**

**Light-Induced Welding of Electrospun Polycaprolactone Nanofibers in a Nonwoven Mat by** **Leveraging the Photothermal Effect of Gold Nanocages**

Haoxuan Li, Yidan Chen, Tong Wu, Wenxia Wang, Haoyan Cheng, Jiajia Xue, and Younan Xia^*^

Dr. H. Li, Dr. T. Wu, Dr. W. Wang, Dr. H. Cheng, Dr. J. Xue, Prof. Dr. Y. Xia

The Wallace H. Coulter Department of Biomedical Engineering, Georgia Institute of Technology and Emory University, Atlanta, GA 30332, USA

Dr. H. Li

State Key Laboratory of Advanced Fiber Materials, College of Materials Science and

Engineering, Donghua University, Shanghai, 201620, P. R. China.

Y. Chen

School of Materials Science and Engineering, Georgia Institute of Technology, Atlanta, GA 30332, USA

Prof. Y. Xia

School of Chemistry and Biochemistry, School of Chemical and Biomolecular Engineering, Georgia Institute of Technology, Atlanta, GA 30332, USA

E-mail: younan.xia@bme.gatech.edu

**Experimental Section**

***Chemicals and materials.*** Silver trifluoroacetate (CF_3_COOAg), poly (vinyl pyrrolidine) (PVP, MW≈55,000), sodium hydrosulfide (NaHS), hydrochloric acid (HCl), gold(III) chloride trihydrate (HAuCl_4_∙3H_2_O), 2,2,2-Trifluoroethanol (TFE), dichloromethane (DCM), and poly(*ε*-caprolactone) (PCL, MW≈80,000) were all obtained from Sigma-Aldrich (St. Louis, MO, USA). Ethylene glycol (EG) was obtained from J. T. Baker (Center Valley, PA, USA).

***Preparation of electrospun nanofibers containing AuNCs.*** We prepared the AuNCs by following a published protocol.^[1]^ The surface of the AuNCs was passivated by PVP. For the PCL fibrous mat, the solution for electrospinning was prepared by dissolving PCL in a solution of TFE at a final concentration of 10% (w/v). Then, the AuNCs in TFE was added to obtain a mixture at a mass percent of 0.01% (w/w). The mixture was loaded into a 5-mL plastic syringe with a 21-gauge needle attached and dispensed using a syringe pump, with an injection rate controlled at 1 mL/h. The distance between the tip of the needle and the collector was about 15 cm and a voltage of 15 kV was applied. Nanofiber mats of varying thickness were obtained by electrospinning for 1, 2, or 3 h. Nanofiber mats with varying AuNC loadings were obtained by adjusting the concentration of AuNCs in the TFE solution from 0.01% to 0.05% and 0.1% (wt./wt.), and the final AuNCs concentration in the PCL nanofiber was calculated at 0.004, 0.018 and 0.037 wt.% by ICP-MS measurement.

***Characterizations*.** The morphologies of the fibrous mats were examined using a SU 8230 field emission scanning electron microscope (FE-SEM) (Hitachi, Japan). Transmission electron microscopy (TEM) images were taken on an HT7700 microscope (Hitachi, Japan) operated at 120 kV. For AuNCs, the sample was prepared by placing a drop of AuNCs (suspended in deionized water) on a carbon-coated copper grid and drying under ambient conditions. For AuNCs/PCL nanofibers, the sample was prepared by collecting the fibers on a carbon-coated copper grid and drying under ambient conditions. The concentration of Au was determined using inductively-coupled plasma mass spectrometry (ICP-MS, NexION 300Q, PerkinElmer, USA). The AuNC-doped nanofiber mats with different AuNC loadings were cut into small circular pieces (1 inch in diameter) and dissolved in 1 mL of DCM overnight under 300 rpm shaking. The AuNCs in the suspension were then collected by centrifugation at 12,000 rpm for 10 min and digested in 2 mL of aqua regia overnight. All samples were diluted with 1% aqueous HNO_3_ to reach the optimal concentration range for ICP-MS measurement. Extinction spectra and transmittances of the AuNCs were recorded using a UV-vis spectrometer (Varian, Cary 50). An 808-nm diode laser coupled to a 100-μm-core nanofiber (Power Technology, USA) was used as the light source. The intensity of the laser was measured to be 0.8 W/cm^2^ with an optical power meter. Fourier transform infrared (FT-IR) spectra in the attenuated total reflection (ATR) mode were obtained using a Varian 640 infrared spectrophotometer (Agilent Technologies, USA). The mechanical properties of the fibrous mats were measured using a WDW-20 micro-controlled electronic universal testing machine (Changchun Science and Technology New Experimental Instrument Co., Ltd., China). The samples were cut into testing strips of 0.5 cm × 2 cm. The samples were tested under uniaxial tension under quasi-static conditions, with a constant tensile speed of 1 mm/s along the direction of motion. The aging test was conducted on QUV/Spray aging test chamber (Q-lab, USA) under ambient conditions.


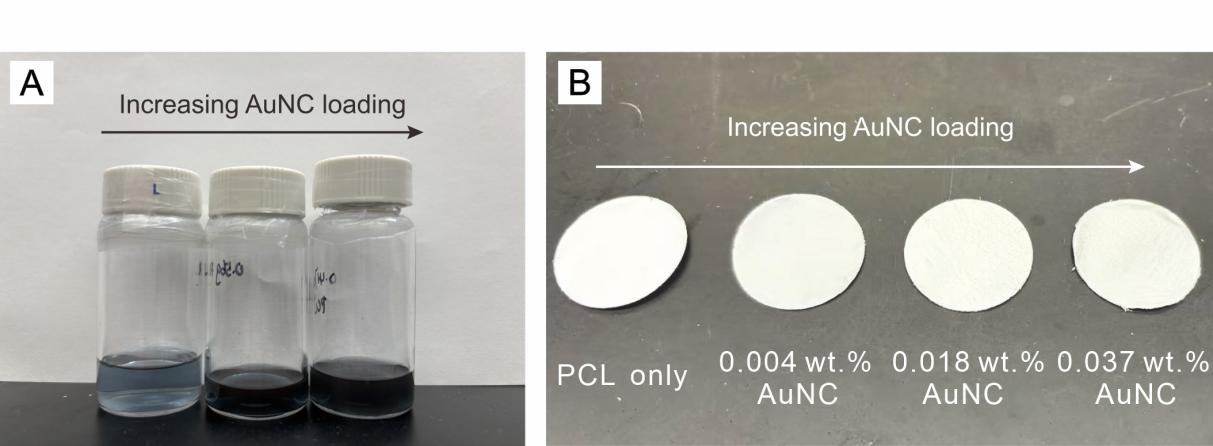


**Figure S1.** Digital photos of the (A) AuNCs in PCL suspension at different AuNC concentrations and (B) the resultant electrospun nanofiber mats.


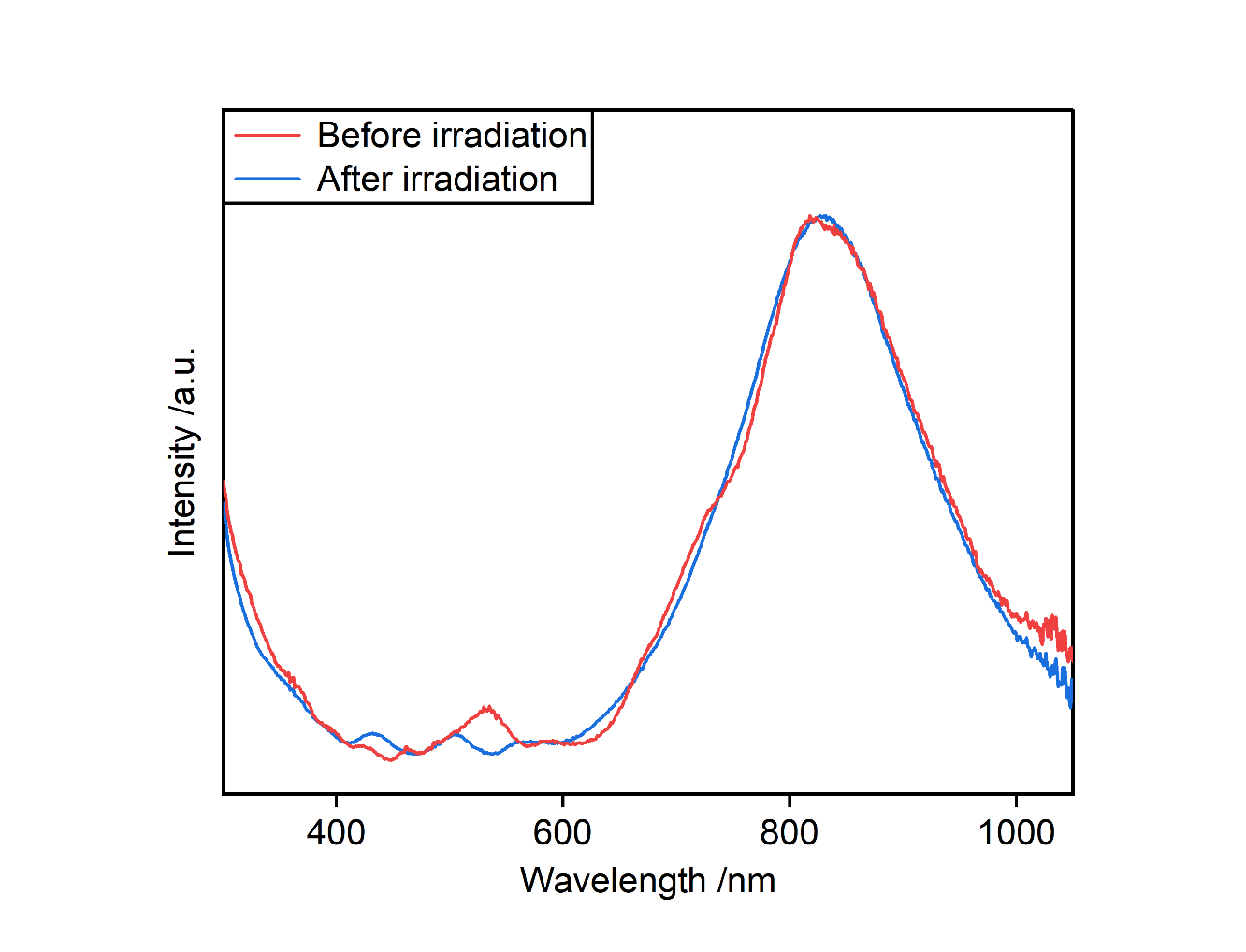


**Figure S2.** UV-vis-NIR absorption spectra recorded from an AuNCs/PCL fibrous mat before and after 808-nm laser irradiation. The spectra are normalized to the maximum peak intensity.


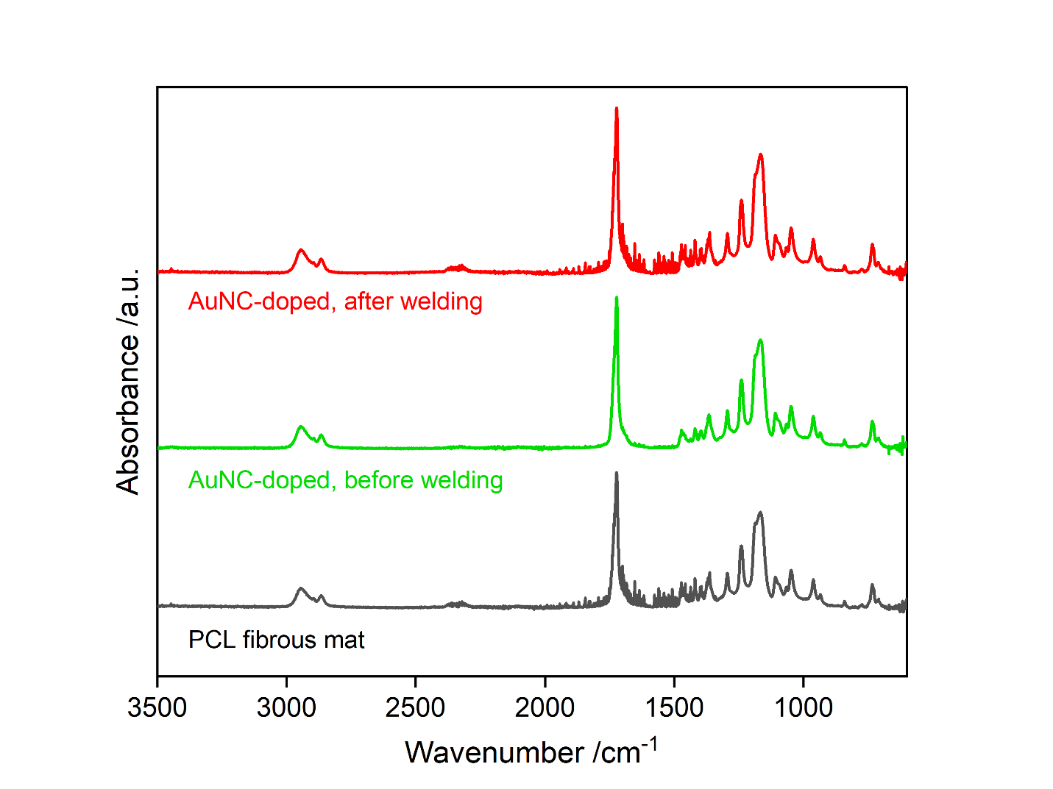


**Figure S3.** FTIR spectra of the AuNC-doped (0.037 wt.%) PCL fibrous mats before and after laser irradiation, in comparison to a pristine PCL fibrous mat.


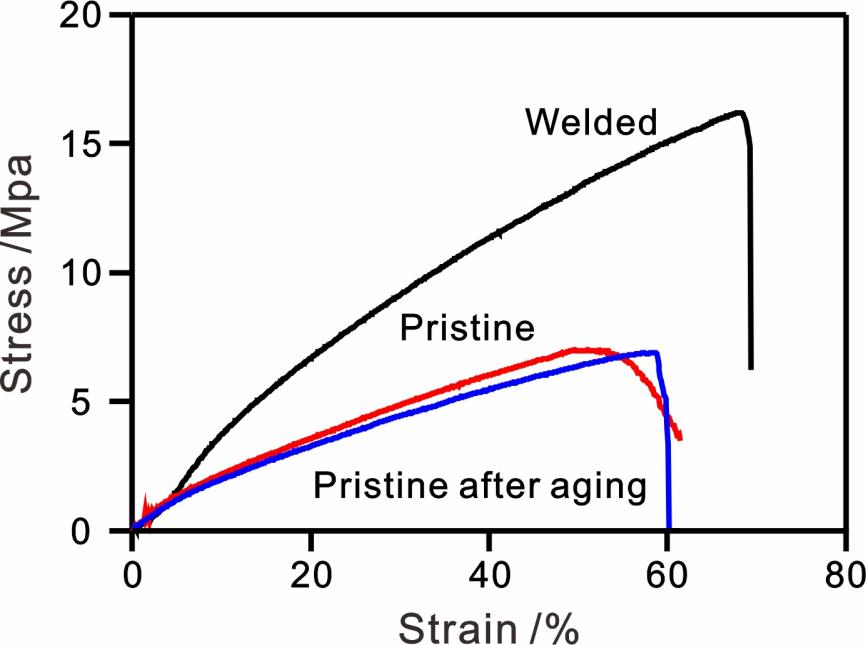


**Figure S4.** Tensile mechanical assessment of the electrospun AuNCs/PCL fibrous mat before and after the treatment with 0.8 W/cm^2^ 808-nm laser irradiation for 2 s, and aging test for 2 weeks.


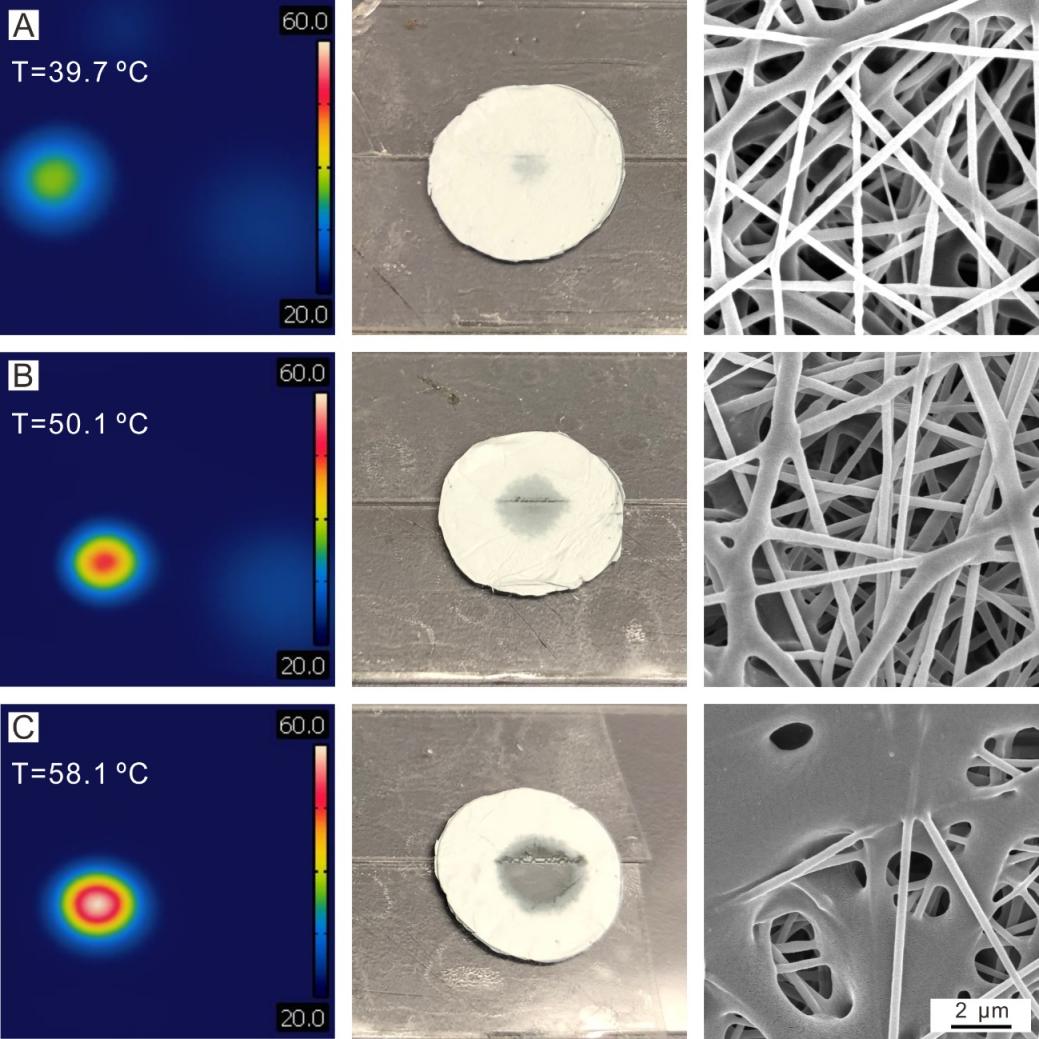


**Figure S5.** Infrared pictures (left), digital photographs (middle), and SEM images (right) showing a fibrous mat containing AuNCs upon irradiation with an 808-nm laser for (A) 1, (B) 3, and (C) 5 s, respectively, at a power density of 0.8 W/cm^2^.


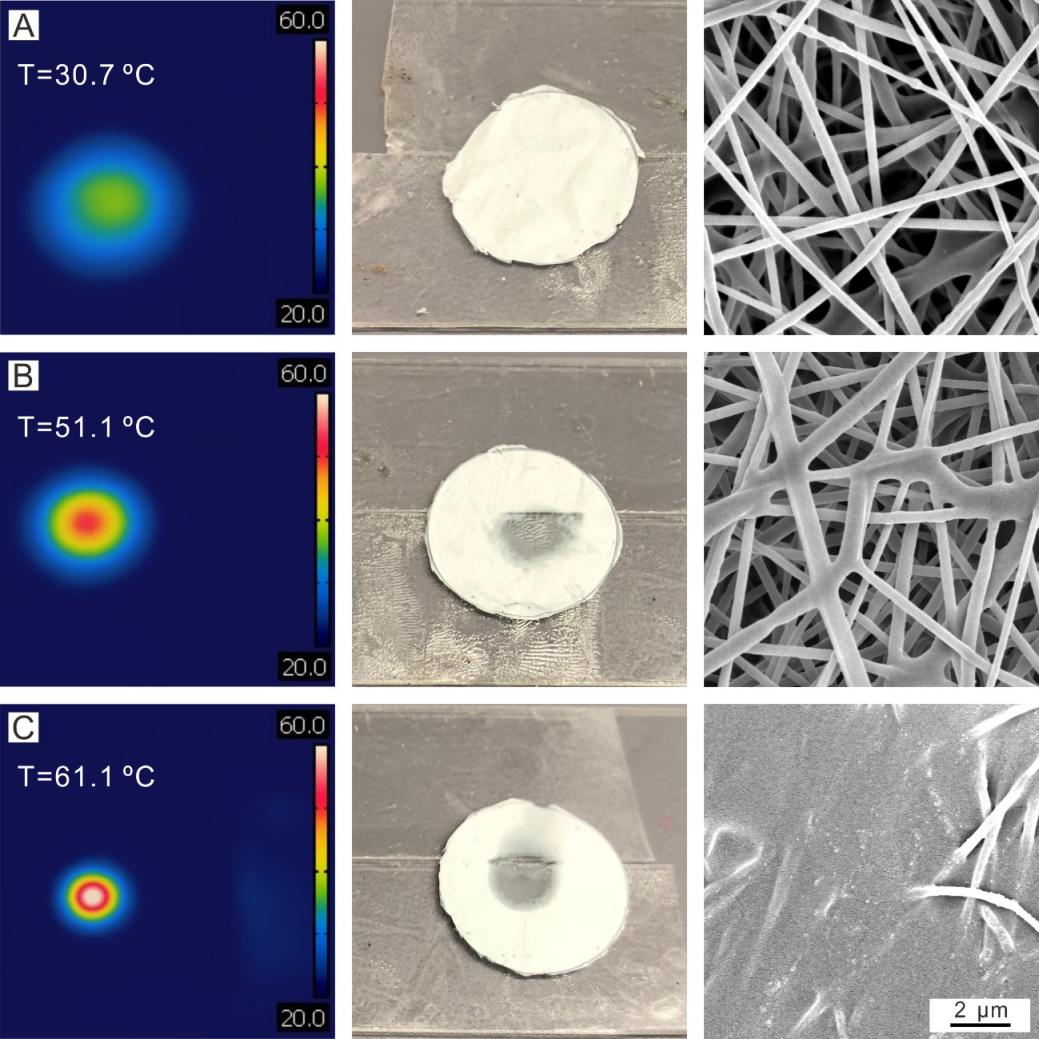


**Figure S6.** Infrared pictures (left), digital photographs (middle), and SEM images (right) showing a fibrous mat containing AuNCs upon irradiation with an 808-nm laser for 3 s at a power density of (A) 0.4, (B) 0.8, and (C) 1.2 W/cm^2^, respectively.


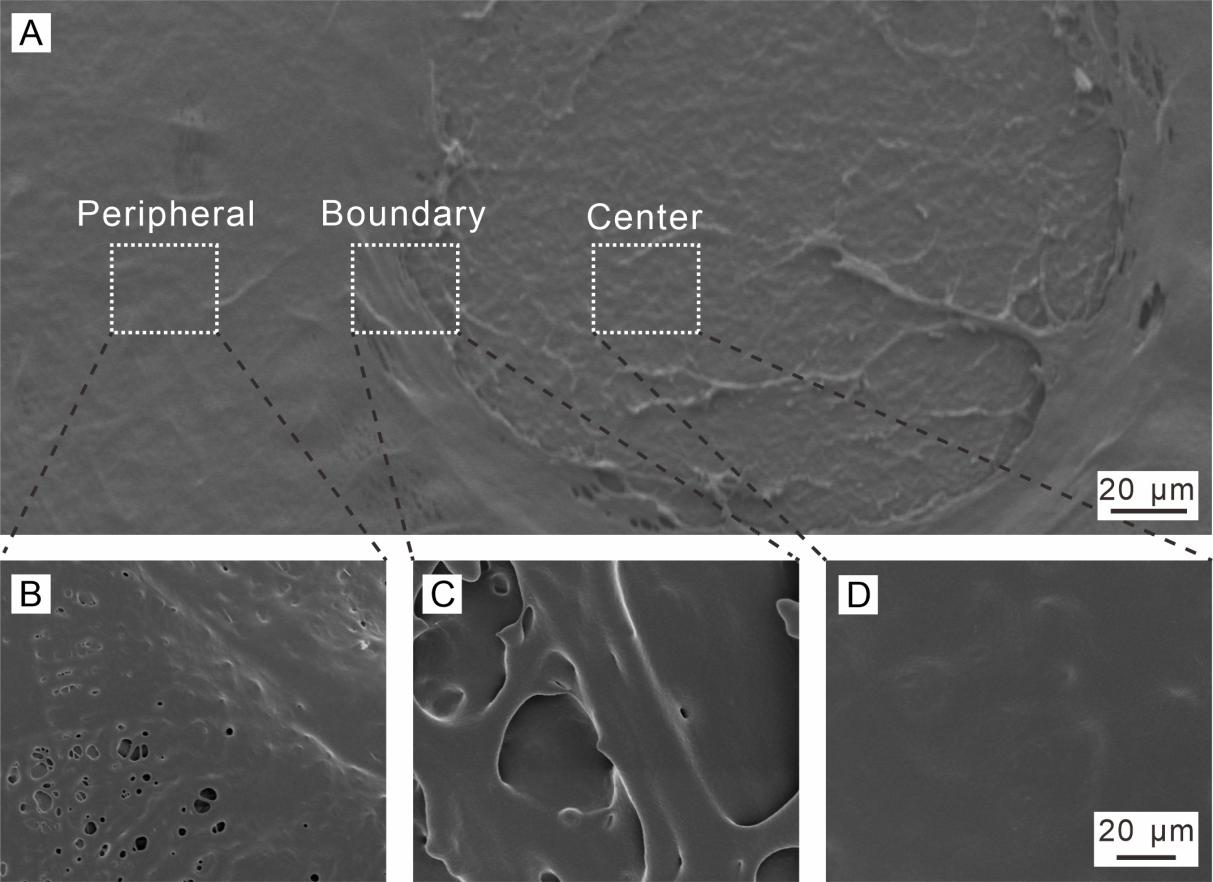


**Figure S7.** SEM images of the top surface of the AuNC-doped fibrous mat after laser irradiation. (A) A zoom-out view showing the irradiated area and the peripheral area. (B-D) zoom-in view of the (B) peripheral, (C) boundary, and (D) center irradiated area corresponding to the marked-out areas in (A). The dotted white line in (A) indicates the laser-irradiated spot.


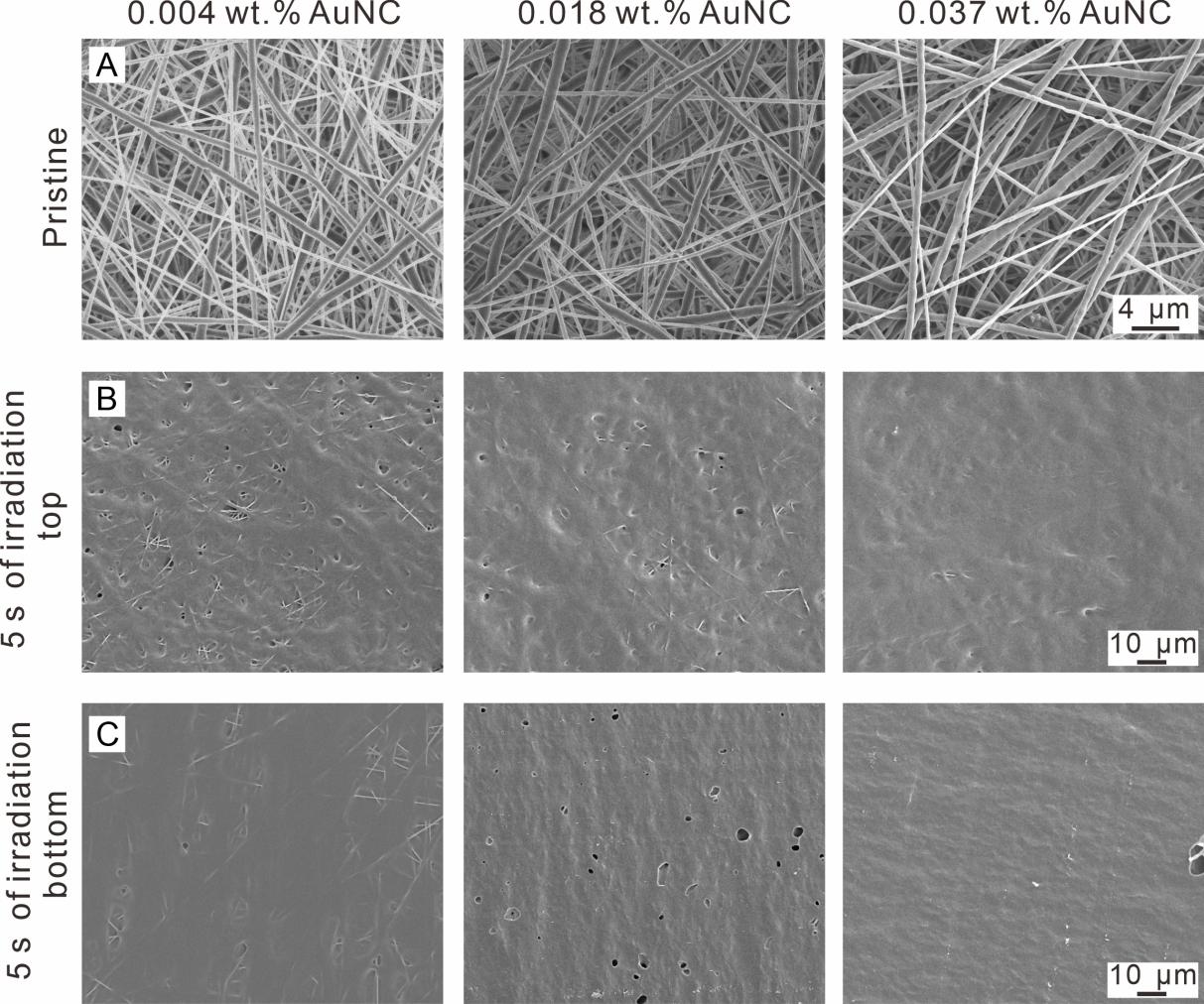


**Figure S8.** SEM images of the AuNCs-doped fibrous mat with different AuNCs loadings. (A) Pristine AuNCs/PCL fibrous mats. (B-C) Welded nanofiber mat after 5s of irradiation with (B) showing the top surface and (C) showing the bottom surface.

**
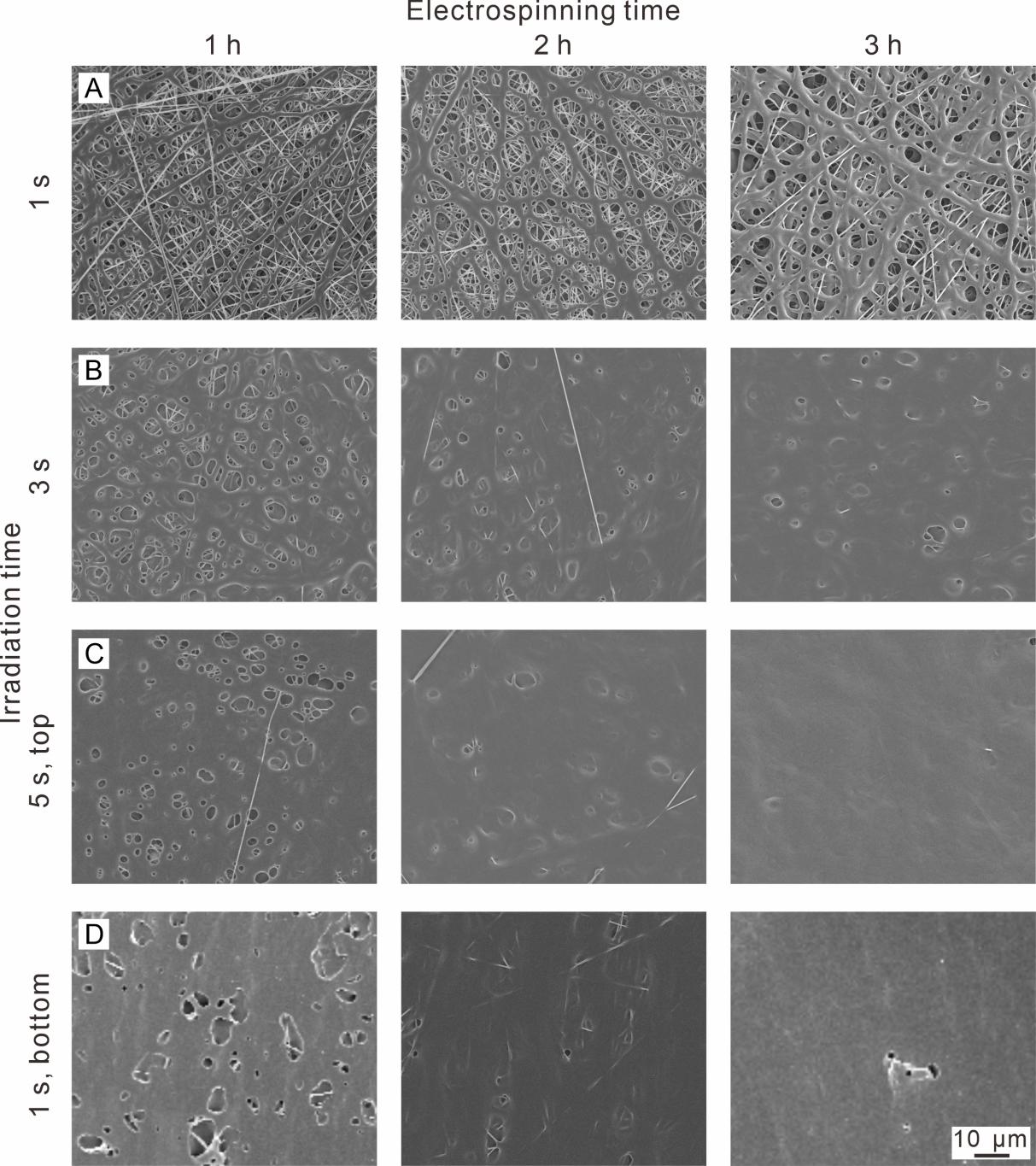
**

**Figure S9.** SEM images showing the AuNC-doped (0.004 wt.%) PCL fibrous mats with different thicknesses, after irradiation by an 808-nm laser at a power density of 0.8 W/cm^2^ for 1, 3, and 5 s, respectively.


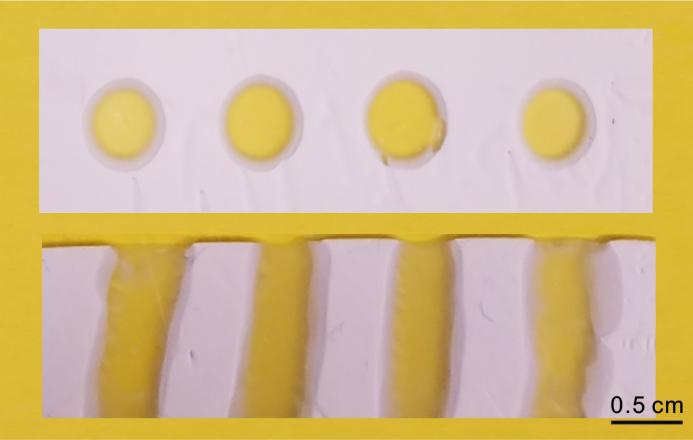


**Figure S10.** Patterns, including dots (top) and stripes (bottom), written on nonwoven mats of AuNC-loaded PCL nanofibers placed on top of a yellow paper through area-selected light welding. The regions irradiated by the laser became transparent due to fusion of the fibers to reveal the color of the paper underneath.

We have conducted ICP-MS measurements on three AuNC-doped PCL fibrous mats with different loadings of AuNCs. The as-obtained loadings were 4.36×10^-3^ wt.%, 1.78×10^-2^ wt.%, and 3.69×10^-2^ wt.%, respectively.

**Table S1. ICP-MS measurements for the mats with three AuNC loadings.**

| Sample | Au Concentration [ppb] | Dilution factor | Total Au [µg] | Sample mass [mg] | Calculated loading wt.% |
| --- | --- | --- | --- | --- | --- |
| 1 | 4.231 | 1:40.54 | 0.343 | 8 | 0.0043 |
| 2 | 3.604 | 1:86.53 | 0.624 | 3.5 | 0.0178 |
| 3 | 3.677 | 1:561.80 | 4.131 | 11.2 | 0.0369 |

**Reference**

[1] S. Skrabalak, L. Au, X. Li, Y. Xia. *Nat. Protocol*, **2007**, *2*, 2182.
